# HDAC1 regulates inflammation and osteogenic differentiation of ankylosing spondylitis fibroblasts through the Wnt-Smad signaling pathway

**DOI:** 10.1186/s13018-022-03224-z

**Published:** 2022-07-06

**Authors:** Yong Zeng, Rui He, Yong Liu, Ting Luo, Qing Li, Yu He, Miao Fang, Taiping Wang

**Affiliations:** Department of Orthopedics, Chengdu Second People’s Hospital, Chengdu, 610017 China

**Keywords:** Ankylosing spondylitis, Fibroblasts, HDAC1, Osteogenic differentiation, Wnt-smad signaling pathway

## Abstract

Ankylosing spondylitis (AS) is a refractory autoimmune disease, whose typical pathology is the development of inflammation to ossification and ankylosis. Histone deacetylase 1 (HDAC1) is considered to be a key factor involved in inflammatory gene transduction, but its role in AS remains unclear. The purpose of this study was to explore the role and possible mechanism of HDAC1 in AS based on the Wnt-Smad pathway. Fibroblasts were isolated from hip synovial tissues of AS patients, adeno-associated virus (AAV) was used to regulate the expression of HDAC1, DKK-1 and SIS3 was used to inhibit Wnt and Smad, respectively. The expressions of Wnt-Smad pathway-related proteins were analyzed by WB, and the TRP ion channel proteins were analyzed by immunofluorescence and WB. The proliferation of AS fibroblasts was detected by CCK-8, the expression of inflammatory cytokines was detected by ELISA, and the effects of HDAC1 on osteogenic differentiation of AS fibroblasts were investigated by alkaline phosphatase (ALP) activity, intracellular calcium concentration, mineralization and osteogenic proteins expressions. Results showed that HDAC1 significantly affected the protein expressions of the Wnt-Smad pathway in AS fibroblasts, and Wnt inhibitor DKK-1 and Smad3 inhibitor SIS3 could significantly reverse the effect of HDAC1 on the Wnt-Smad pathway. In addition, HDAC1 significantly activated the TRP ion channel and promoted the proliferation, inflammatory response and osteogenic differentiation of AS fibroblasts. DKK-1 or SIS3 treatment significantly inhibit the effect of HDAC-1 on AS fibroblasts, suggesting that the Wnt-Smad pathway is involved in the regulation of AS by HDAC1. In conclusion, HDAC1 promotes the proliferation, inflammatory response and osteogenic differentiation of AS fibroblasts through the Wnt-Smad pathway.

## Introduction

Ankylosing spondylitis (AS) is an autoimmune disease mainly characterized by chronic inflammation of axial joints and tendon-ligament bone attachment points [1]. It mainly affects the spine and the sacroiliac joint, and can be accompanied by systemic multi-system damage [2]. With the progression of the disease, patients develop spinal movement limitation, rigidity, and spinal joint destruction, which in severe cases can lead to disability and seriously affect the quality of life of patients [3]. The pathogenesis of AS is complex, genetic factors, the interaction between microbe and host, hormone level differences and abnormal immune responses have all been proved to be involved in the pathogenesis of AS [4]. In-depth understanding of the pathogenesis of AS and search for new therapeutic targets are the focus of current research.

Histone acetylation is a heritable process of changing gene expression that does not involve DNA sequence changes, and its status is regulated by histone acetyltransferase (HAT) and histone deacetylases (HDAC) [5]. Histone acetylation and deacetylation are important processes that regulate gene expression in inflammatory responses [6]. Studies have shown that HDAC is a key factor involved in inflammatory gene transduction and cell proliferation, and HDAC inhibitors have a good therapeutic potential for inflammatory diseases [7]. AS is an autoimmune disease, which usually develops from the initial stage of inflammation to the stage of ossification and rigidity [8], suggesting that HDAC may play an important role in AS. An imbalance between HAT and HDAC activities has been observed in AS patients, where TNF-α production can be regulated by HDAC inhibitor therapy [9]. HAT/HDAC ratios in AS also increased significantly during anti-TNF-α therapy [10]. As a type of HDAC, HDAC1 is a key player in T-cell-mediated arthritis [11]. Studies have shown that HDAC1 is highly expressed in synovial tissues of rheumatoid arthritis, which can promote synovial cell hyperplasia and inflammation [12, 13]. In addition, inhibition of HDAC1 inhibits inflammation and bone loss in arthritis [14], and inhibitors targeting HDAC1 may be useful in the treatment of arthritis. However, the role and possible mechanism of HDAC1 in AS remain to be further studied.

Numerous studies have shown that inflammation is associated with new bone formation, and inflammation can trigger new bone formation [15]. In this process, TNF-α, a key factor of the AS inflammatory cell network, plays an important role together with Wnt and Smad pathways. Wnt signaling pathway plays an important role in a variety of cellular processes [16], and abnormal regulation of this pathway is considered to be a key factor in the pathogenesis of AS [17]. In addition, the Smad signaling pathway is an important intracellular transforming growth factor signaling pathway and also plays a key role in bone development and regeneration [18]. Wnt and Smad signaling pathways are believed to play an important role in bone formation and homeostasis [19]. Our previous study also confirmed that there was a cross-association of Wnt and Smad pathways in AS fibroblasts. However, whether they are involved in the regulation of AS by HDAC1 remains unclear.

This study was designed to clarify the effects of HDAC1 on Wnt and Smad signaling pathways in AS, and further explore the effect of HDAC1 on inflammatory response and osteogenic differentiation of AS fibroblasts based on Wnt-smad pathways.

## Material and methods

### Cell culture

Hip synovial tissues were collected from AS patients (no restriction on age and gender) undergoing total hip arthroplasty in the Chengdu Second People's Hospital, and hip synovial tissues from patients with femoral neck fracture (sampling time to trauma less than 24 h) were selected as control. This experiment was approved by the Chengdu Second People's Hospital (No. 2022089), under the ethical guidelines of the declaration of Helsinki. The fibroblasts were obtained from tissue samples by the tissue block culture method. Briefly, the tissue samples were rinsed with phosphate buffer solution (PBS) to remove the fat, blood and muscle on the tissue surface, then the cleaned tissue was cut into 1 mm^3^ piece and inoculated in the Dulbecco's modified eagle medium (DMEM) containing 20% fetal bovine serum (FBS) and 1% penicillin/streptomycin at 37 °C 5% CO_2_ cell incubator. The culture medium was replaced once every 7 days after the cells around the tissue blocks crawled out and gradually shortened to once every 4 days with the growth of cells. Passage culture was performed when the cells were 80% full of the bottom of the flask. The passage cells were cultured at 37 °C 5% CO_2_ cell incubator, and the third-generation cells in the logarithmic growth phase were used in this experiment.

### Cell transfection

HDAC1 adeno-associated virus (AAV-HDAC1) was packaged by Shanghai Genechem Co., LTD (China) in strict accordance with the adenovirus operating instructions and stored in virus tubes at − 80 °C. Fibroblasts were inoculated into 6‑well plates at a density of 4 × 10^5^ cells per well and cultured at 37 °C overnight. AAV-HDAC1 or AAV-negative control (AAV-NC) was then co-incubated with cells using HitransGP infection enhancer solution (Shanghai Genechem Co., LTD, China). After 6 h of infection, the culture medium containing the virus was sucked out and replaced with a fresh medium, and the culture was continued at 37 °C. Follow-up experiments were performed 48 h after AAV-HDAC1 or AAC-NC treatment.

### Osteogenic differentiation

Fibroblasts were inoculated into 6‑well plates at a density of 1 × 10^5^ cells per well. After cells reached 80% confluence, the original medium was discarded, and cells were cultured in osteogenic induction medium (DMEM containing 10% FBS, 0.1 μM dexamethasone, 30 mM vitamin C and 10 mM β‑glycerophosphate) [20] for 14 days to induce the differentiation of cells into osteoblast. The medium was replaced every 3 days.

### Alizarin red staining

After the induction of osteogenic differentiation, the cells were fixed with 10% paraformaldehyde for 10 min, washed with PBS 3 times, and incubated with 1% Alizarin Red S (Sigma-Aldrich, USA) solution for 10 min at room temperature. After staining, cells were washed with PBS 3 times and observed under a microscope (Nikon Corporation, Japan), and quantitative statistics were performed with ImageJ software version 1.8.0 (National Institutes of Health).

### Alkaline phosphatase (ALP) activity assay

Cells were collected and the protein contents were measured using a BCA Protein Assay Kit (Beijing Solarbio Science and Technology Co., Ltd.). ALP activity, an early marker of osteogenic differentiation, was analyzed by an ALP assay kit (WLA064, Wanleibio) according to the manufacturer’s instructions. ALP activity was normalized to the protein content.

### Immunofluorescent staining

The cells were fixed at 4% paraformaldehyde prepared in cytoskeletal buffer for 10 min at 37 °C [21] and washed with PBS 3 times. Then, the cells were blocked by bovine serum albumin for 1 h. Then, the primary anti-TRPC1 (1:1000; cat. no. #DF12783, Affinity Biosciences) and anti-TRPV4 (1:1000; cat. no. #DF8624, Affinity Biosciences) were incubated at 4 °C overnight, and the fluorescent secondary antibody Alexa Fluor 488-labeled Goat Anti-Rabbit IgG (H + L) (1:500; cat. no. A0423; Beyotime Biotechnolog) was used to incubate at room temperature and away from light for 1 h. Finally, the 4',6-diamidino-2-phenylindole (DAPI, Sigma, USA) was used for re-staining the nucleus for 5 min and observed under THUNDER Imager Tissue (Leica, Germany).

### Western blot analysis

Total cellular proteins were obtained by lysing cells in Cell lysis buffer for Western and IP (cat. no. P0013; Beyotime) and the proteins were determined by BCA Protein Assay Kit (cat. no. P0009; Beyotime). Protein samples were denatured, separated via SDS-PAGE, and subsequently transferred onto PVDF membranes (cat. no. ISEQ00010; Sigma-aldrich). The membranes were blocked for 1 h at room temperature in TBST containing 5% skimmed milk, then incubated overnight at 4 °C with anti-HDAC1 (1:1000; cat. no. #AF6433), anti-Wnt1 (1:1000; cat. no. #AF5315), anti-Smad3 (1:1000; cat. no. #AF6362), anti-Phospho (p)-Smad3 (1:1000; cat. no. #AF3362), anti-β-catenin (1:1000; cat. no. #AF6266), anti-GSK-3β (1:1000; cat. no. #AF5016), anti-Axin (1:1000; cat. no. #DF6978), anti-TRPC1 (1:1000; cat. no. #DF12783), anti-TRPV4 (1:1000; cat. no. #DF8624), anti-BMP2 (1:1000; cat. no. #AF5163), anti-Osterix (1:1000; cat. no. #DF7731), anti-Osteopontin (OPN; 1:1000; cat. no. #AF0227), anti-β‑actin (1:4000; cat. no. #AF7018) antibodies (all purchased from Affinity Biosciences; USA). After washing 3 times with TBST, the membranes were incubated with HRP-conjugated secondary antibody (1:4000; cat. no. #S0001; Affinity) for 2 h at room temperature. Proteins on the membranes were visualized with an enhanced chemiluminescence detection kit (Bio-Rad Laboratories, Inc.) using ChemiScope 6000 (Clinx Science Instruments Co., Ltd.). Densitometry was performed using ImageJ software version 1.8.0 (National Institutes of Health).

### Cell viability assay

Cells were inoculated into 96-well culture plates at a density of 5 × 10^3^ per well and treated with DKK-1 and/or SIS3 for 24 h. Cell viability was detected using a Cell Counting Kit-8 (CCK-8; Beyotime Institute of Biotechnology, China) at 37 °C for 1 h. The absorbance was measured at 450 nm using a microplate spectrophotometer (cat. no. 1681150; Bio-Rad Laboratories, Inc.) and cell viability was calculated as follows:

$${\text{Cell viability }}\left( \% \right) \, = {{\left( {A_{{{\text{experimental}}}} - A_{{{\text{blank}}}} } \right)} \mathord{\left/ {\vphantom {{\left( {A_{{{\text{experimental}}}} - A_{{{\text{blank}}}} } \right)} {\left( {A_{{{\text{control}}}} - A_{{{\text{blank}}}} } \right)}}} \right. \kern-\nulldelimiterspace} {\left( {A_{{{\text{control}}}} - A_{{{\text{blank}}}} } \right)}}\; \times \;100$$

### Enzyme-linked immunosorbent assay

The expression of inflammatory cytokines IL-6, IL-1β and TNF-α in AS fibroblast were detected by ELISA. All operations are carried out in strict accordance with the product instructions of the kit (EIAAB SCIENCE INC, Wuhan, China), and repeated at least three times for each sample.

### Statistical analysis

All experimental data were analyzed using SPSS 20.0 software, and expressed as mean ± standard deviation (SD). The differences among groups were statistically compared by one-way analysis of variance (ANOVA) and Tukey HSD *post-hoc*, and P < 0.05 was considered as statistically significant. All experiments were independently repeated at least three times.

## Results

### Effects of HDAC1 on Wnt and Smad signaling pathways in AS fibroblasts

**Fig. 1:**
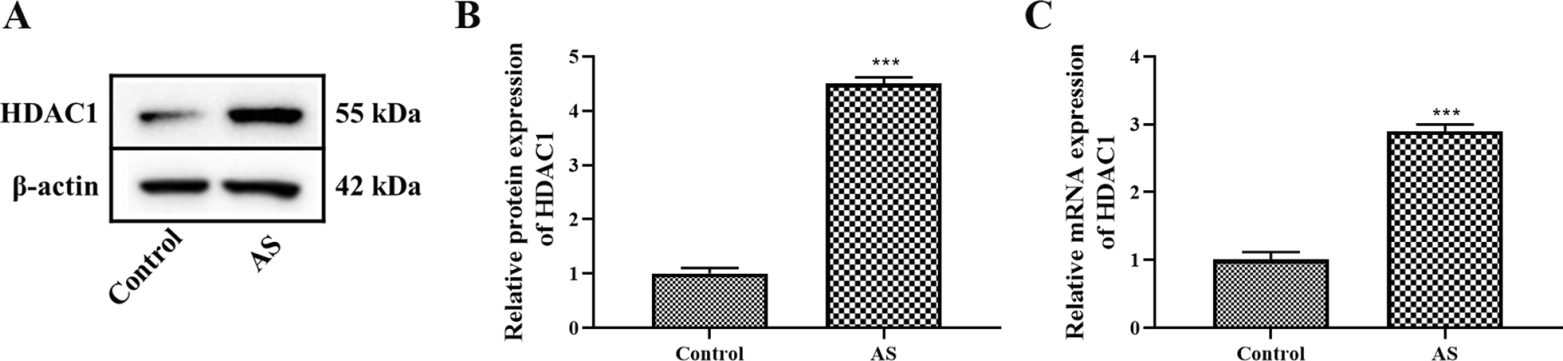
Expression of HDAC1 in AS fibroblasts. **A** The protein expression of HDAC1 in AS fibroblasts was detected by western blot analysis. **B** Densitometry analysis of protein expression. **C** Gene expression of HDAC1 in AS fibroblasts was detected by RT-PCR analysis. Data were shown as mean ± SD. ^*^P < 0.05, ^**^P < 0.01 and ^***^P < 0.001, compared with the control group

**Fig. 2:**
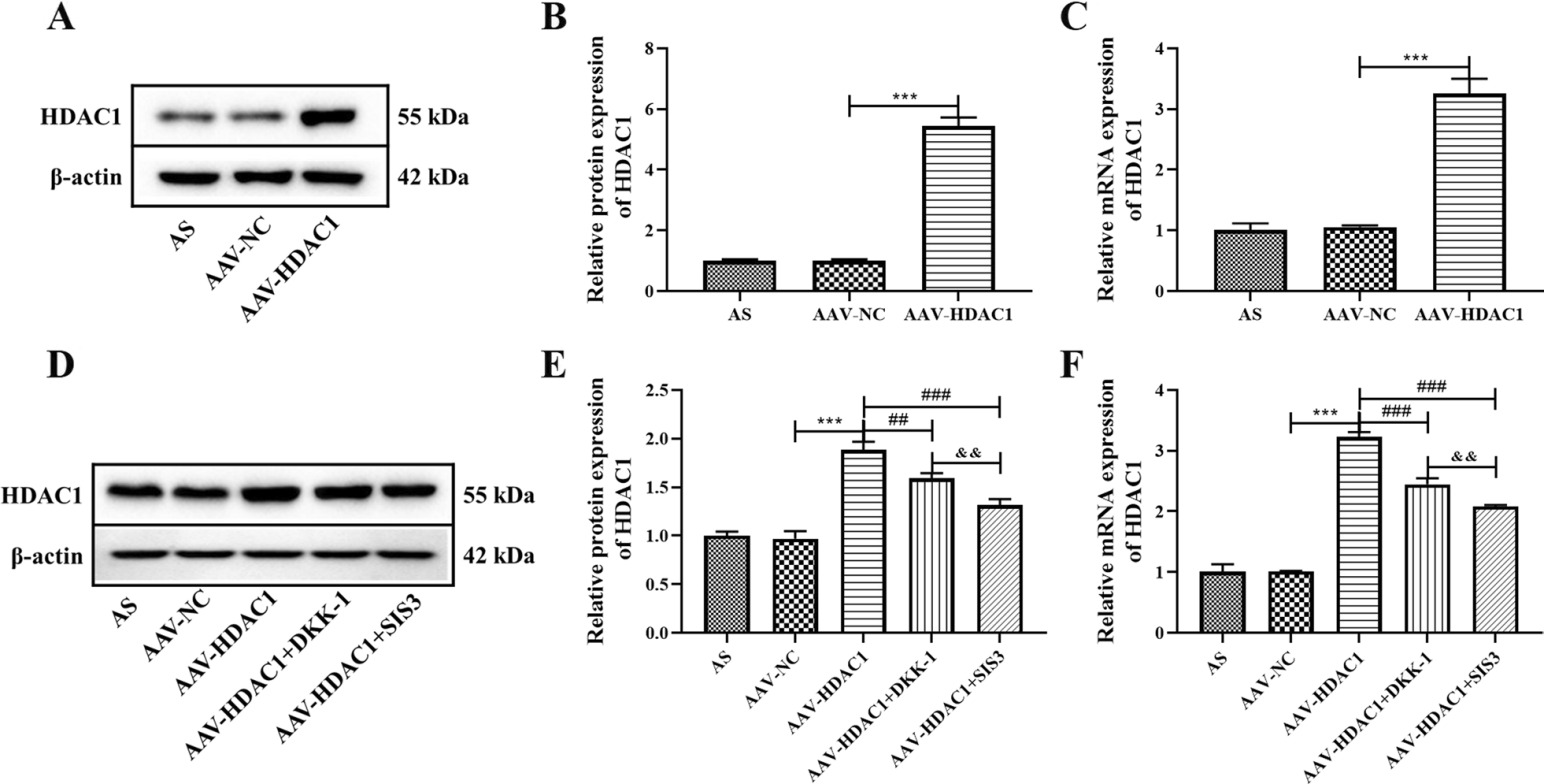
Proteins expression of HDAC1 in AS fibroblasts after different treatment. **A** The expression of HDAC1 was overexpressed by AAV-HDAC1, and the protein expression was detected by western blot analysis. **B** Densitometry analysis of protein expression. **C** Gene expression of HDAC1 in AS fibroblasts after AAV-HDAC1 treatment was detected by RT-PCR analysis. **D** Protein expressions of HDAC1 in AS fibroblasts transfected with AAV-HDAC1 after DKK-1 and SIS3 treatment were detected by western blot analysis. **E** Densitometry analysis of protein expression. **F** Gene expressions of HDAC1 in AS fibroblasts were detected by RT-PCR analysis. Data were shown as mean ± SD. ^*^P < 0.05, ^**^P < 0.01 and ^***^P < 0.001, compared with the AAV-NC group. ^#^*P* < 0.05, ^##^*P* < 0.01 and ^###^*P* < 0.001, compared with the AAV-HDAC1 group. ^&^*P* < 0.05, ^&&^*P* < 0.01 and ^&&&^*P* < 0.001, compared with the AAV-HDAC1 + DKK-1 group

Studies have shown that HDAC1 is highly expressed in synovial tissues of rheumatoid arthritis, which can promote synovial cell hyperplasia and inflammation [12, 13]. In this study, the expression of HDAC1 in AS fibroblasts was also analyzed, and the results confirmed that the protein and gene expression of HDAC1 in AS fibroblasts was significantly higher than that in the control group (Fig. 1A–C *P* < 0.05). Then, HDAC1 was overexpressed by adeno-associated virus (AAV-HDAC1), and the Wnt inhibitor DKK-1 and the Smad3 inhibitor SIS3 were used to treat AS fibroblasts transfected with AAV-HDAC1 to analyze the effects of HDAC1 on Wnt and Smad signaling pathways in AS fibroblasts. The experiment was divided into five groups: AS group, AAV-NC group, AAV-HDAC1 group, AAV-HDAC1 + DKK-1 group (500 ng/ml recombinant human Dkk1, R&D Systems) and AAV-HDAC1 + SIS3 group (3 µM, R&D Systems). The expression of HDAC1 in AS fibroblasts was detected by WB and RT-PCR, and the results showed that the protein and gene expressions of HDAC1 were significantly increased after AAV-HDAC1 treatment (Fig. 2A–C *P* < 0.05). Compared with the AAV-HDAC1 group, the protein and gene expressions of HDAC1 were significantly decreased in the AAV-HDAC1 + DKK-1 and AAV-HDAC1 + SIS3 group (Fig. 2D–F *P* < 0.05). It was noteworthy that the effect of SIS3 on HDAC1 is more obvious than that of DKK-1 (*P* < 0.05).

**Fig. 3:**
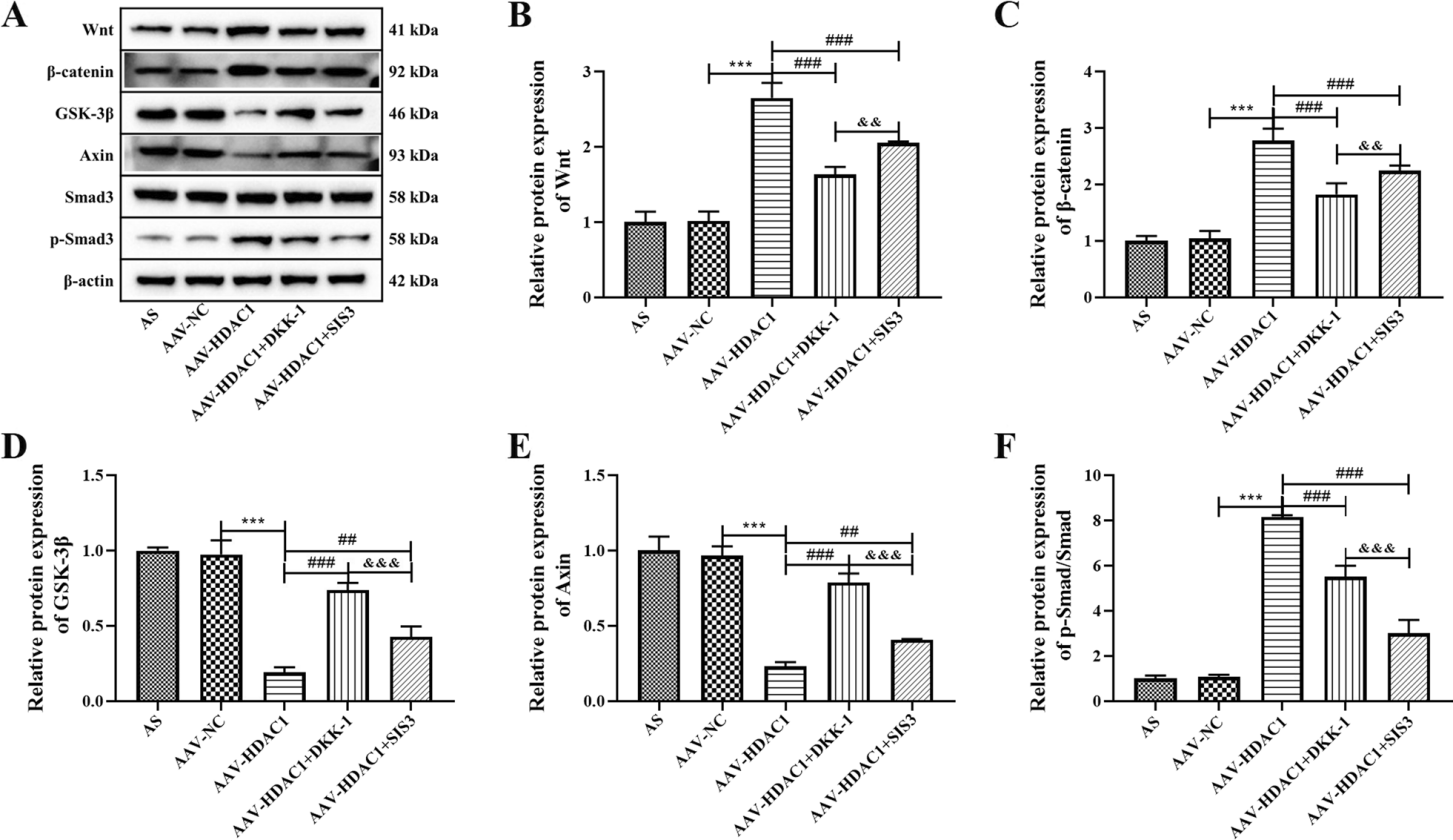
Proteins expression of Wnt and Smad signaling pathways in AS fibroblasts after different treatment. **A** Protein expressions of Wnt, β-catenin, GSK-3β, Axin, Smad3 and p-Smad3 in AS fibroblasts were detected by western blot analysis. **B** Densitometry analysis of Wnt protein expression. **C** Densitometry analysis of β-catenin protein expression. **D** Densitometry analysis of GSK-3β protein expression. **E** Densitometry analysis of Axin protein expression. **F** Densitometry analysis of p-Smad/Smad protein expression. Data were shown as mean ± SD. ^*^P < 0.05, ^**^P < 0.01 and ^***^P < 0.001, compared with the AAV-NC group. ^#^*P* < 0.05, ^##^*P* < 0.01 and ^###^*P* < 0.001, compared with the AAV-HDAC1 group. ^&^*P* < 0.05, ^&&^*P* < 0.01 and ^&&&^*P* < 0.001, compared with the AAV-HDAC1 + DKK-1 group

In addition, the effects of HDAC1 on Wnt and Smad signaling pathways in AS fibroblasts were analyzed by WB. The results showed that compared with the AAV-NC group, the expressions of Wnt, β-catenin and p-Smad/Smad were significantly increased and the expressions of GSK-3β and Axin were significantly decreased after AAV-HDAC1 transfected, suggesting that HDAC1 significantly affected Wnt and Smad pathways in AS fibroblasts (Fig. 3, *P* < 0.05). In addition, AAV-HDAC1 + DKK-1 and AAV-HDAC1 + SIS3 significantly reversed the increase of Wnt, β-catenin and p-Smad/Smad protein expression, and decrease of GSK-3β and Axin protein expression induced by AAV-HDAC1 (Fig. 3, *P* < 0.05). It was further suggested that Wnt and Smad pathways are involved in HDAC1 regulation of AS fibroblasts.

### Effects of HDAC1 on TRP channels in AS fibroblast

**Fig. 4:**
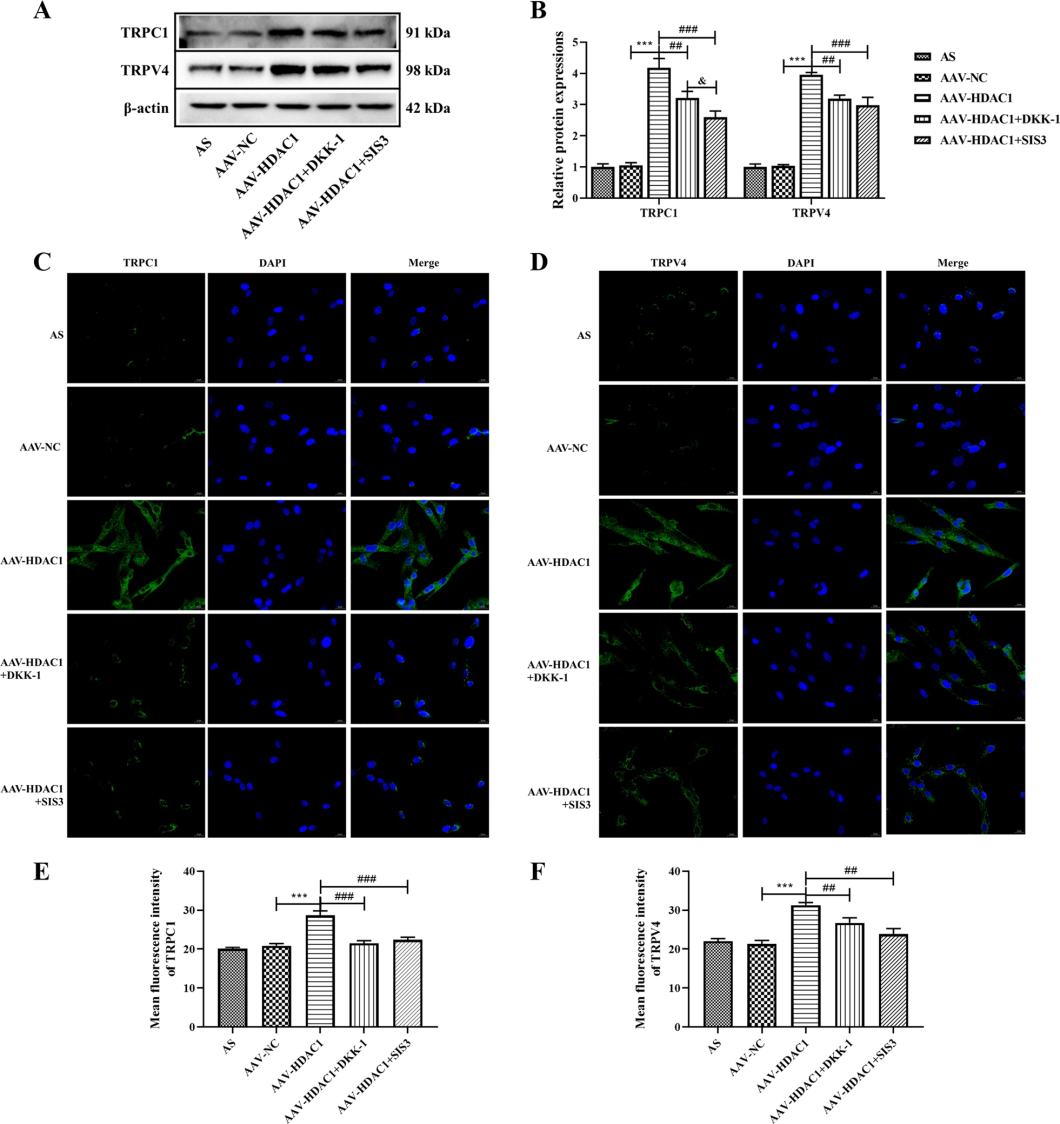
Expression of TRPC1 and TRPV4 in AS fibroblasts after different treatment. **A** Protein expressions of TRPC1 and TRPV4 in AS fibroblasts were detected by western blot analysis. **B** Densitometry analysis of protein expression. **C** Representative images showing immunofluorescence staining of TRPC1. Magnification, × 400. **D** Representative images showing immunofluorescence staining of TRPV4. Magnification, × 400. **E** Mean fluorescence intensity of TRPC1. **F** Mean fluorescence intensity of TRPV4. Data were shown as mean ± SD. ^*^P < 0.05, ^**^P < 0.01 and ^***^P < 0.001, compared with the AAV-NC group. ^#^*P* < 0.05, ^##^*P* < 0.01 and ^###^*P* < 0.001, compared with the AAV-HDAC1 group. ^&^*P* < 0.05, ^&&^*P* < 0.01 and ^&&&^*P* < 0.001, compared with the AAV-HDAC1 + DKK-1 group

AS is an autoimmune disease, in which TRP channels are involved in a variety of cellular functions of the immune system and play an important role in many diseases [22]. Here, we analyzed the effect of HDAC1 on the TRP ion channel in AS fibroblast, WB results showed that HDAC1 significantly increased the protein expression of TRPC1 and TRPV4, suggesting that HDAC1 significantly affected the TRP ion channel in AS fibroblasts. In addition, DKK-1 and SIS3 significantly reduced the protein expressions of TRPC1 and TRPV4 in AAV-HDAC1-transfected cells, and reversed the activation of TRP ion channels in AS fibroblasts by HDAC1 (Fig. 4A and B, *P* < 0.05). The immunofluorescence results were consistent with the WB analysis results (Fig. 4C–F), all of which indicated that HDAC1 significantly regulated the expression of TRP ion channel proteins in AS fibroblast, and that Wnt and Smad pathways might be involved.

### Effects of HDAC1 on inflammation and osteogenic differentiation of AS fibroblasts

**Fig. 5:**
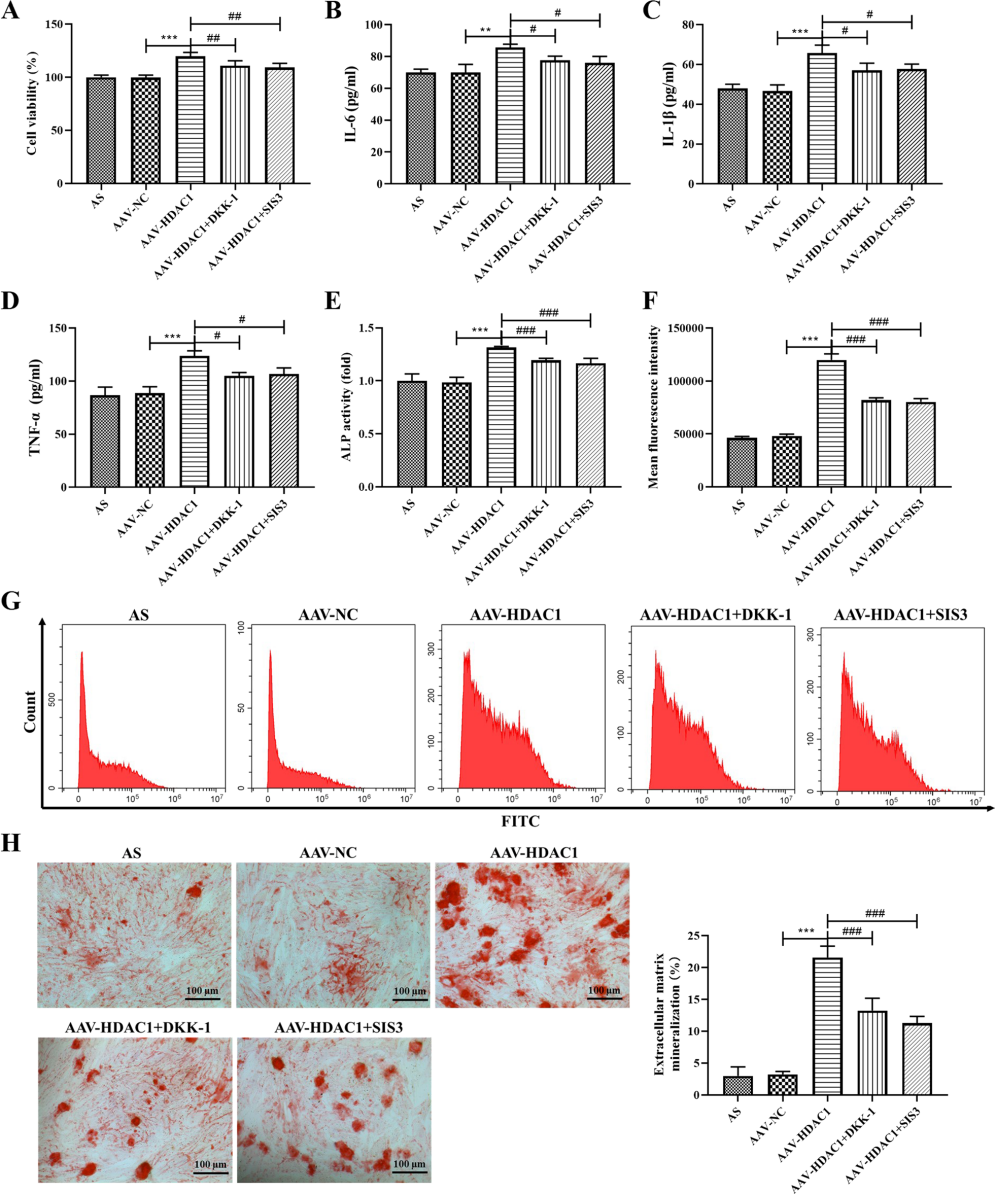
Effects of HDAC1 on inflammation and osteogenic differentiation of AS fibroblasts. **A** Cells viability was detected by CCK-8 assay. **B** IL-6 expression in AS fibroblasts was detected by ELISA. **C** IL-1β expression in AS fibroblasts was detected by ELISA. **D** TNF-β expression in AS fibroblasts was detected by ELISA. **(E)** ALP activity of AS fibroblasts. **F** Mean fluorescence intensity of calcium ions. **G** Intracellular calcium concentration was detected by flow cytometry. **H** Representative images showing Alizarin red staining. Magnification, × 100. Data were shown as mean ± SD. ^*^P < 0.05, ^**^P < 0.01 and ^***^P < 0.001, compared with the AAV-NC group. ^#^*P* < 0.05, ^##^*P* < 0.01 and ^###^*P* < 0.001, compared with the AAV-HDAC1 group

**Fig. 6:**
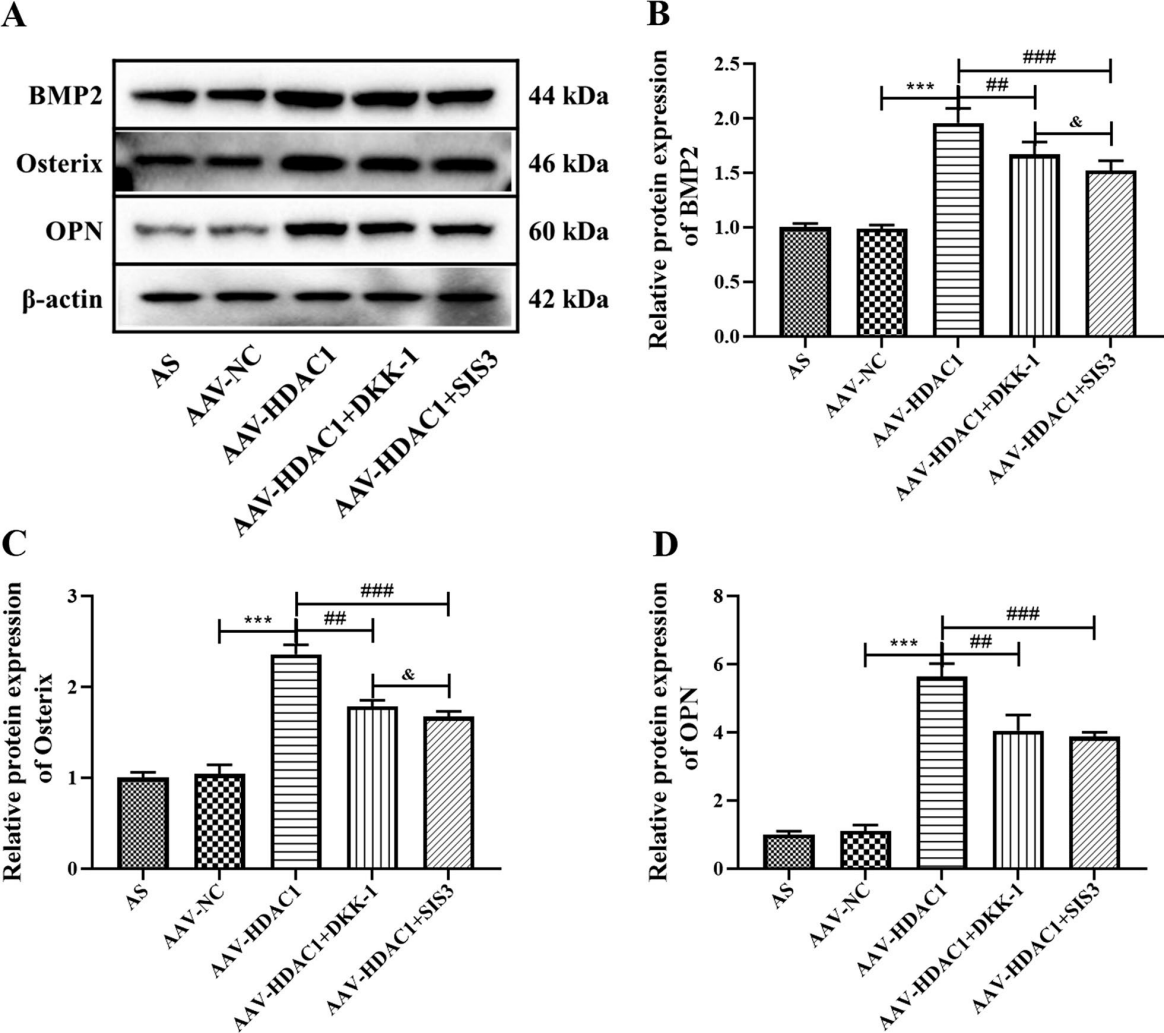
Protein expression of osteogenic differentiation related proteins in AS fibroblasts after different treatment. **A** Protein expressions of BMP2, Osterix and OPN in AS fibroblasts were detected by western blot analysis. **B** Densitometry analysis of BMP2 protein expression. **C** Densitometry analysis of Osterix protein expression. **D** Densitometry analysis of OPN protein expression. Data were shown as mean ± SD. ^*^P < 0.05, ^**^P < 0.01 and ^***^P < 0.001, compared with the AAV-NC group. ^#^*P* < 0.05, ^##^*P* < 0.01 and ^###^*P* < 0.001, compared with the AAV-HDAC1 group. ^&^*P* < 0.05, ^&&^*P* < 0.01 and ^&&&^*P* < 0.001, compared with the AAV-HDAC1 + DKK-1 group

To further explore the role of HDAC1 in AS, we analyzed the cell viability, expression of inflammatory cytokines and osteogenic differentiation ability of AS fibroblasts. CCK-8 results showed that HDAC1 significantly increased the cell activity of AS fibroblasts and promoted cell proliferation, while DDK-1 and SIS3 could significantly reverse the increased viability of AS fibroblasts induced by AAV-HDAC1 (Fig. 5A, *P* < 0.05), suggesting that HDAC1 may promote AS fibroblasts proliferation through Wnt and Smad pathways. In addition, HDAC1 significantly increased the expression of IL-6, IL-1β and TNF-α, while DKK-1 or SIS3 significantly reversed the increase of inflammatory cytokines induced by HDAC1 (Fig. 5B–D, *P* < 0.05). And HDAC1 significantly increase the expression of ALP and the concentration of intracellular calcium ions, while DKK-1 or SIS3 treatment significantly reduced the expression of ALP and the concentration of intracellular calcium ions increased by AAV-HDAC1 (Fig. 5E–G, *P* < 0.05). Also, alizarin red staining showed that osteogenic differentiation ability was significantly increased in the AAV-HDAC1-transfected cells, which was reversed by DKK-1 or SIS treatment (Fig. 5H). Further analysis of osteogenic differentiation-related proteins showed that HDAC1 could significantly increase the protein expression of bone morphogenetic protein 2 (BMP2), Osterix and Osteopontin (OPN), and DKK-1 or SIS3 could significantly reduce the protein expression of BMP2, Osterix and OPN in the AAV-HDAC1-transfected cells (Fig. 6, *P* < 0.001). All the results confirmed that HDAC1 could positively regulate the osteogenic differentiation of AS fibroblasts through Wnt and Smad pathways.

## Discussion

AS is a chronic and progressive inflammatory disease, whose typical pathological changes are from the initial inflammation to ossification. If the inflammation progress is not controlled in time, it may eventually lead to disability [23]. At present, the pathogenesis of ankylosing spondylitis remains unclear. HDAC1 has been confirmed to be highly expressed in rheumatoid arthritis and is thought to play an important role in inflammatory diseases [24]. In this study, we found that HDAC1 can significantly regulate Wnt and Smad signaling pathways in AS fibroblasts, and Wnt inhibitor DKK-1 and Smad inhibitor SIS3 can reverse this effect. In addition, HDAC1 can significantly activate TRP ion channels, promote cell proliferation, inflammatory response and osteogenic differentiation of AS fibroblasts. DKK-1 and SIS3 also reverse the effects of HDAC1 on AS cell activity, inflammatory response and osteogenic differentiation. All studies confirmed that HDAC1 was involved in the progression of AS through Wnt and Smad pathways.

Inflammation, bone destruction and ectopic ossification are three characteristic pathological links in the course of AS, and ectopic ossification is the key to disability of AS [4]. Heterotopic ossification of AS mainly occurs in the spine and connective tissue around joints, and fibroblasts are an important part of these surrounding tissues, which can transform to osteogenic type under certain conditions [25, 26]. Under the stimulation of some special cytokines, AS fibroblasts proliferate abnormally, activate osteogenic potential, secrete bone matrix, and eventually deposit enough calcium particles in collagen to be fixed AS bone cells [27]. Our previous study has found that fibroblasts cultured in *vitro* from AS patients have strong osteogenic differentiation ability. This study continues to explore the pathological mechanism of AS from the perspective of fibroblasts by using AS fibroblasts.

HDAC1 is the first discovered HDAC that plays an important role in gene regulation, cell cycle and cell differentiation [28]. It has been confirmed that dysregulation of HDACs in peripheral blood mononuclear cells, macrophages and fibroblast-like synoviocytes in rheumatoid arthritis is associated with higher disease activity [13, 29, 30]. The anti-inflammatory effects of various non-selective HDAC inhibitors have been described in various arthritis models, suggesting that they may be used to treat inflammatory and autoimmune diseases [31, 32]. The present study showed that HDAC1 significantly increased the expression of inflammatory cytokines IL-6, IL-1β and TNF-α in AS fibroblasts, and Wnt inhibitor DKK-1 or Smad inhibitor SIS3 reversed the inflammatory response caused by HDAC1, confirming that HDAC1 may promote the inflammatory response of AS fibroblasts through the Wnt and Smad pathways. Wnt and Smad pathways are believed to play important roles in many cellular processes, including osteogenic differentiation [33, 34]. Our previous study confirmed that Wnt and Smad signaling pathways synergistically inhibit proliferation and osteogenic differentiation of AS fibroblasts. In this study, HDAC1 significantly increased the expression of Wnt, β-catenin and the ratio of p-Smad/Smad, and decreased the expression of GSK-3β and Axin in AS fibroblasts, suggesting that Wnt and Smad pathways are also involved in the regulation of AS fibroblasts by HDAC1.

Calcium is the most abundant mineral in the body and plays an important role in the regulation of physiological processes [35, 36]. Continuous calcium influx across cell membranes is important for both lymphocyte activation and initiation of innate and adaptive immune responses [37]. Transient receptor potential (TRP) ion channels are common calcium channels that are activated by environmental stimuli and also respond to endogenous factors and messengers produced during tissue injury and inflammation [38]. As molecular sensors, TRP channels are widely expressed in the entire immune system, responsible for detecting the external world and internal environment, and are associated with a variety of physiological processes and diseases [39]. The present study found that HDAC1 significantly increased the expression of TRPC1 and TRPV4, and activated TRP ion channels, and also increased the intracellular calcium concentration of AS fibroblasts. In addition, ALP is a nonspecific phosphomonoesterase that plays an important role in calcium deposition, and is an important marker of osteogenic activity [40]. It is commonly used to determine the ability of early osteogenic differentiation [41]. This study showed that HDAC1 significantly increased the mineralization and ALP activity of AS fibroblasts, and this effect was reversed by DKK-1 or SIS3, confirming that HDAC1 may promote osteogenic differentiation of AS fibroblasts through Wnt and Smad pathways.

To further explore the role of HDAC1 in the osteogenic differentiation of AS fibroblasts, the expression of osteogenic marker proteins BMP2, Osterix and OPN were detected by WB analysis. BMP2, a member of the transforming growth factor-β superfamily, plays an important role in many stages of bone regeneration, inducing osteogenic differentiation and endochondral ossification [42]. Osterix, also known as Sp7, is another important osteogenic specific transcription factor that is expressed only in bone tissue cells and is essential for osteoblast differentiation and bone formation [43]. And OPN is a phosphorylated, vulcanized glycoprotein capable of absorbing hydroxyapatite and plays an important role in the mineralization of connective tissue, which is related to bone resorption and formation [44]. In this study, HDAC1 significantly increased the expression of osteogenic differentiation-related proteins BMP2, Osterix and OPN in AS fibroblasts, while DKK-1 and SIS3 reversed this effect, further suggesting that HDAC1 can promote the differentiation of AS fibroblasts into osteoblasts through Wnt and Smad pathways.

## Conclusion

In conclusion, this study found that HDAC1 significantly affected the expression of Wnt and Smad signaling pathway proteins in AS fibroblasts. In addition, HDAC1 can significantly activate TRP ion channels and promote the proliferation, inflammatory response and osteogenic differentiation of AS fibroblasts. The Wnt inhibitor DKK-1 and smad3 inhibitor SIS3 significantly reversed the regulation of HDAC1 on AS, suggesting that HDAC1 was involved in the disease progression of AS through the Wnt and Smad pathways.

## Data Availability

The datasets used and/or analyzed during the current study are available from the corresponding author on reasonable request.
